# Current Concepts in the Biopsy of Musculoskeletal Tumors

**DOI:** 10.1155/2013/538152

**Published:** 2013-06-05

**Authors:** Costantino Errani, Francesco Traina, Fabrizio Perna, Carlotta Calamelli, Cesare Faldini

**Affiliations:** Orthopaedic Service, Rizzoli Institute, Rizzoli-Sicilia, Strada Statale 113 Km, 246-90011 Bagheria, Italy

## Abstract

In the management of bone and soft tissue tumors, accurate diagnosis, using a combination of clinical, radiographic, and histological data, is critical to optimize outcome. On occasion, diagnosis can be made by careful history, physical examination, and images alone. However, the ultimate diagnosis usually depends on histologic analysis by an experienced pathologist. Biopsy is a very important and complex surgery in the staging process. It must be done carefully, so as not to adversely affect the outcome. Technical considerations include proper location and orientation of the biopsy incision and meticulous hemostasis. It is necessary to obtain tissue for a histological diagnosis without spreading the tumor and so compromise the treatment. Furthermore, the surgeon does not open compartmental barriers, anatomic planes, joint space, and tissue area around neurovascular bundles. Nevertheless, avoid producing a hematoma. Biopsy should be carefully planned according to the site and definitive surgery and should be performed by an orthopedic surgeon with an experience in musculoskeletal oncology who will perform the definitive surgery. Improperly done, it can complicate patient care and sometimes even eliminate treatment options. Different biopsy techniques are suitable: fine-needle aspiration, core-needle biopsy, and incisional biopsy. The choice of biopsy depends on the size, the location of the lesion, and the experience of the pathologist.

## 1. Introduction

Proper diagnosis is imperative for the appropriate management of musculoskeletal tumors, and biopsy is a critical step in the diagnosis of bone and soft tissue tumors. The goal of biopsy is to obtain diagnostic tissue while minimizing morbidity, limiting potential tumor spread, and avoiding interference with future treatments [1].

A poorly performed biopsy could become an obstacle to proper diagnosis and may have negative impact on future treatments [2]. Mankin et al. [3] evaluated a study on 597 patients who underwent a biopsy for bone and soft tissue sarcomas. The rate of major errors in diagnosis was 13.5%, and the rate of complications was 15.9%, and unnecessary amputations were performed in 3% of these patients. These events occurred more frequent, when the biopsy was performed in a referring institution, rather than in an oncology center.

As a general rule, unless proven otherwise, all lesions should be treated as if they are malignant, and the biopsy has to be delayed until the imaging is complete [4]. In fact, the staging of the lesion helps determine the exact anatomic approach to the tumor and specifies the region of the tumor that represents the underlying disease [2]. As an alternative to open biopsy, percutaneous core-needle biopsy techniques have been developed [5].

The literature contains a number of controversies regarding the diagnostic yield of these techniques. The purpose of this paper is to illustrate the current concepts in the biopsy of musculoskeletal tumors. The diagnostic accuracy should be the most important parameter in determining the choice of the biopsy technique. Biopsy tissue can be obtained through a fine-needle aspiration, a core-needle biopsy, or an incisional biopsy [1].

## 2. Biopsy Techniques

General guidelines regarding the biopsy of musculoskeletal tumors are independent of the technique. (a) Because bone and soft tissue tumors are usually heterogeneous, multiple samples are required to establish a diagnosis. This procedure does not promote metastatic dissemination but can spread tumor cells locally and so increase the risk of local recurrence. (b) For this reason, it must be assumed that the biopsy tract may be contaminated and thus should be resected during the definitive surgery. Therefore, it is mandatory that the biopsy must be made with the planned surgical incision site, so that it will be included with the surgical specimen. (c) Moreover, the biopsy tract must not violate more than one anatomic compartment and must be away from the neurovascular bundle [2].

### 2.1. Fine-Needle Aspiration Biopsy

The incidence of false negative is high. Even when positive, the diagnosis cannot always be precise. The main limitation is that it does not permit the evaluation of tissue architecture, and in addition, cytologic specimens are not always adequate for ancillary, cytogenetic, molecular, or immunohistochemical studies. The advantage is that it is a relatively atraumatic procedure. Moreover, it has low cost ($1060) and morbidity [1]. It can be used for local or distant recurrence where the cytology findings can be compared with the prior histology specimens [6].

### 2.2. Core-Needle Biopsy

The false negative rate is lower. It is useful in lesions where the histological study of small sample is sufficient to confirm the clinical-imaging appearance. The architecture of tissue is preserved; thus, it is usually possible not only the histological diagnosis and the grade tumor, but also allows an immunohistochemical or molecular analysis. The advantages are low risk of contamination and minimally invasive procedure for the patient [7]. In deep musculoskeletal tumors coordination with ultrasound or CT guidance is an easy and safe procedure, and can be useful to increase the biopsy accuracy [8]. This kind of biopsy can be performed in an outpatient clinic with the use of local anesthesia; so the cost ($1106) and time are less than open biopsy [1, 9]. A disadvantage is a lower rate of accuracy compared with open biopsy. In the absence of adequate tissue, open biopsy is required [10].

### 2.3. Incisional Biopsy

**Figure 1:**
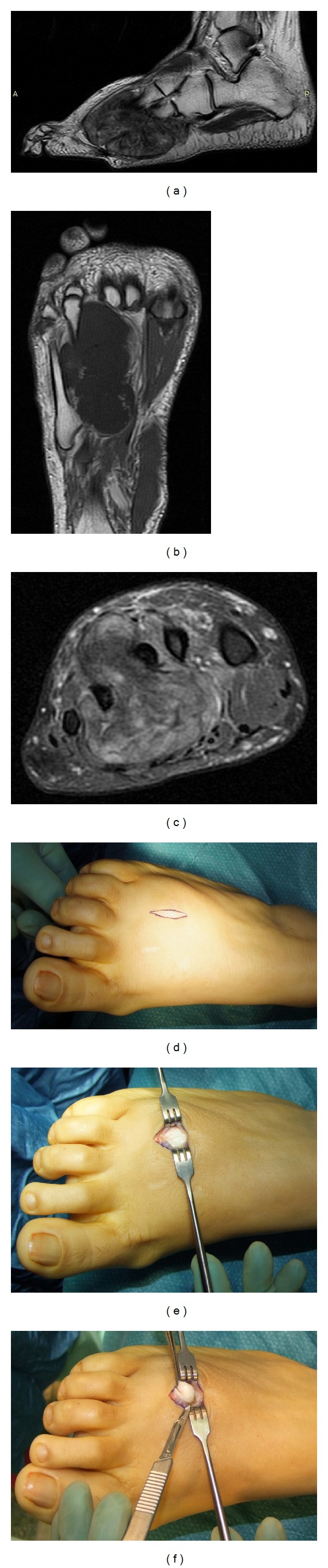
Sagittal MRI T2 (a), coronal MRI T1 (b), and axial MRI fat sat (c) scans of the right foot show heterogeneous intratumoral hypointensive signal on T1 and hyperintensive signal on T2. Incisional biopsy is made by (d) a small longitudinal incision between the second and third metatarsals; then (e) once the tumor capsule is found, (f) the tissue sampling is done with cold knife. Diagnosis: soft tissue chondroma.

It is preferred in the “difficult cases” (Figure 1). When accurate histological study on large specimens to obtain diagnosis is a predicate of preoperative chemotherapy or radiotherapy, then an open biopsy is best, and when a core biopsy comes out inadequate [11]. It can be used in association with frozen section analysis to ensure that diagnostic material has been obtained, and, if a benign diagnosis is done, an excision is even indicated. The biopsy must be done by extensile exposure which usually means a longitudinal approach, along the line of the incision which will be used for the definitive surgical excision. It is mandatory to use the smallest incision that is compatible with obtaining adequate specimen. Transverse incisions are contraindicated because they require a wider soft tissue resection at the time of definitive surgery [2].

It is very important to establish absolute hemostasis to avoid hematoma and the risk of local tumor spread in the hematoma. Any hematoma around a tumor should be considered contaminated. Large hematoma may dissect the soft and subcutaneous tissues and contaminate the entire extremity, making limb salvage surgery impossible [2]. If a tourniquet is used, it must be removed before wound closure to allow accurate haemostasis.

A drain is usually not used, but in the uncommon case where a drain is required, it should exit along and near the skin incision (~1 cm). The drain path is considered contaminated and has to be excised with the surgical specimen, in the same manner of the biopsy tract [2].

Disadvantages associated with this procedure have included spillage of tumor cells and wound complications. Wound complications and inappropriate situation of the incision may also compromise local treatment [12]. Open surgical biopsy may enlarge the extent of sarcoma resection and compromise function. If the diagnosis is benign, then there are no functional sacrifices [13]. The cost ranges from $4321.25 to $7234.00 [1].

### 2.4. Bone Biopsies

Biopsy is a crucial step in the diagnosis of bone tumors. It might be omitted only in the case of clinically and radiologically unambiguous benign lesions, such as chondroma, osteochondroma, osteoid osteoma, simple bone cyst, fibrous dysplasia, or nonossifying fibroma. The different procedures such as fine-needle aspiration, core needle, and incisional biopsy have the objective to gain a representative tissue sample with minimal trauma [14]. The shortest distance to the lesion is not necessarily the optimal route [4]. In general, the biopsy should be carefully planned according to the site of the definitive surgery. Improperly done, it can complicate patient care and sometimes even eliminate treatment options. 

Open biopsy has for a long time been considered the gold standard. This procedure has to be performed by an expert orthopaedic oncologist in line with the planned resection and according to sarcoma principles [14]. The samples are taken from the periphery of the tumor due to the frequent presence of central necrosis [14]. It is reasonable to assume that the risk of local contamination is higher in comparison to needle biopsy, and this is related to the width of the biopsy tract and adequacy of hemostasis. For this reason, open biopsy is usually indicated when, after a percutaneous biopsy, the diagnosis is inconclusive or does not correlate with the clinical presentation and radiologic findings [2].

As a rule, the extraosseous component of malignant bone tumors is as representative of the tumor as is the bony component and should have a biopsy. Violating the cortex of a bone predisposes the patient to a pathologic fracture and is recommended only if there is no extraosseous extension of the tumor [2]. When a purely, intraosseous bone lesion is having a biopsy, make a cortical window and pay attention to its shape. Clark et al. reported that an oblong hole with rounded ends afforded the greatest residual strength of bone compared to rectangular holes with square or rounded corners. In addition, they found that increasing the width of the hole caused a significant reduction in strength, but increasing the length did not [15].

**Figure 2:**
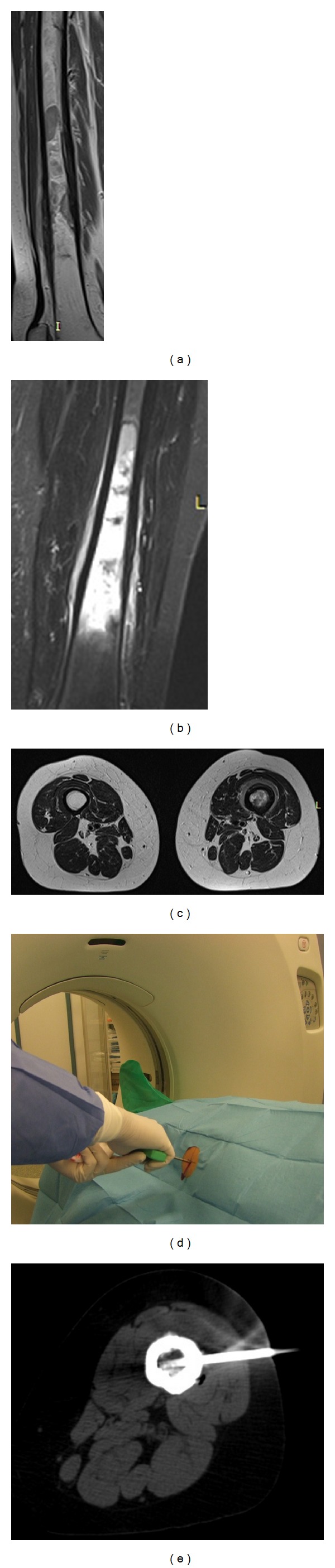
Sagittal MRI T1 (a), coronal MRI STIR (b), and axial MRI T2 (c) scans of the right thigh show heterogeneous intratumoral hypointensive signal on T1 and hyperintensive signal on T2 in the diaphysis of the femur. An 8-gauge core-needle bone biopsy inserted under CT guide (d) into marrow of the right femur (e). Diagnosis: Lymphoma.

Recent studies increasingly suggest similar diagnostic accuracy for percutaneous techniques compared to open biopsy [14]. In addition, percutaneous biopsies have a lower risk of complications than surgical incisional biopsies, 0 to 10% compared with up to 16%, respectively [4]. The main complications are bleeding, nerve apraxia, and infection [4]. During percutaneous biopsy, the use of CT guidance or fluoroscopy guidance provides excellent spatial localization of the lesion (Figure 2). A multiple core can be obtained through a single bone window, thereby reducing the risk of iatrogenic fracture. 

Pohlig et al. [14] retrospectively compared core-needle biopsy versus open biopsy in 48 cases of bone tumors. The diagnostic accuracy rate was 100% for core-needle biopsy and 93.3% for incisional biopsy. However, there was not a statistically significant difference (*P* > 0.05). These findings correlate well with the recent literature, indicating no difference in accuracy between core and open biopsy [5, 16].

In case of deep lesions, for example, in the pelvis or spine, needle biopsies are challenging procedures. In this case, the aid of CT guidance has further increased the accuracy, reduced complications, and therefore has now become the procedure of choice [17].

### 2.5. Soft Tissue Biopsies

The most definitive diagnostic procedure for most soft tissue masses is a biopsy. However, not all soft tissue lesions require treatment or a biopsy. There are instances where the clinical and imaging features are so typical that the biopsy may not be required, and it is possible to identify the nature of the soft tissue mass: lipoma, hemangioma, and neurofibroma for benign soft tissue tumors, ganglions and popliteal cysts, myositis ossificans, and PVNS for pseudotumors lesions [18]. A biopsy is indicated whenever a mass has biological activity, and further surgical or medical treatment will be based on that result. Virtually, all soft tissue masses more than 3 cm in diameter or growing lesions should be biopsied [19].

Biopsy should be planned and performed after imaging studies are completed [19]. The biopsy site should be directly over the tumor, at the point where the lesion is closest to the surface, and raising of flaps or disturbance of tissue planes superficial to the tumor should be avoided [20]. Biopsy tract has to be performed in the region of the surgical incision at the time of definitive surgery. It is better to take tissue at the periphery of the lesion, where there are viable cells. On the contrary, necrosis and so nondiagnostic tissue are usually present within the tumor. A minimum of three core samples are usually required [4].

Excision of the biopsy tract and the tumor as one piece with adequate surgical margins is necessary at the time of definitive surgery. This requires the biopsy incision to be placed in a position that can be safely excised en bloc with the resected specimen [19].

The first objective of a biopsy is to obtain diagnostic material. This can be accomplished with fine-needle aspiration, core needle, or open surgical biopsy [19]. Diagnostic difficulty is usually associated with myxoid and round cell neoplasms. In addition, the paraspinal anatomic location had the lowest diagnostic accuracy rate [19].

Incisional biopsy has long been the gold standard for soft tissue tumors diagnosis, with an accuracy of 94% to 99%; however, it is expensive and carries a complication rate of up to 16%, including hematoma, tumor spread, and wound problems that may interfere with future treatments. Therefore, less invasive techniques have emerged [1, 21–23].

Fine-needle aspiration is usually accepted for documentation of metastases and local recurrences, especially if prior samples are available for comparison [21]. In fact, although this technique distinguishes mesenchymal from metastatic tumors, malignant from benign lesions, and high from low grade sarcomas, it is unable to precisely subtype sarcomas [21]. In regard to this technique, the literature reports a wide range of sensitivities (86%–100%), specificities (36%–100%) and diagnostic accuracies (21.9%–98%). However, these studies usually exclude nondiagnostic samples, reducing the reliability of results [1]. Ng et al. [21] retrospectively examined the diagnostic accuracy of 432 fine-needle aspirations performed in soft tissue masses of extremity. They reported that the nature of the lesion was indeterminate or the sample was inadequate in 8.1% of the cases. Subtyping and grading for malignant lesions were 77.2% and 95.2% accurate, respectively. One-fourth of all patients required a secondary biopsy before a definitive treatment [21].

Core biopsy has evolved as an alternative to fine-needle aspiration. This technique improves diagnosis of histologic subtype and grade compared with fine-needle aspiration [1]. The sensitivity ranges from 81.8% to 100%, specificity from 91% to 100%, and diagnostic accuracy from 72.7% to 100%. In addition, the complication rate is only a 0.1% to 1.1% [1]. However, as with fine-needle aspiration, these studies often excluded nondiagnostic samples, which may falsely elevate accuracy rates [1].

Though much literature exists regarding the diagnostic yield of biopsy techniques individually, however, there are only two studies that compared accuracy of biopsy techniques in the same soft tissue tumor [1, 6].

Yang and Damron [6] compared fine-needle aspiration and core biopsy in the diagnosis of the same soft tissue mass and found core biopsy to be more accurate than fine-needle aspiration, 83% and 64% accuracy, respectively.

Kasraeian et al. [1] prospectively studied 57 patients with soft tissue masses, performing fine-needle aspiration, followed by core-needle biopsy, followed by incisional biopsy of the same mass. Incisional biopsy was 100% accurate on all accounts. Fine-needle aspiration and core-needle biopsy had an overall accuracy of 75.4% and 80.7%, respectively. However, in regard to determining the exact diagnosis, fine-needle aspiration had a 33.3% accuracy, and core-needle biopsy had a 45.6% accuracy. Therefore, they recommend open biopsy in the diagnosis of soft tissue masses [1].

**Figure 3:**
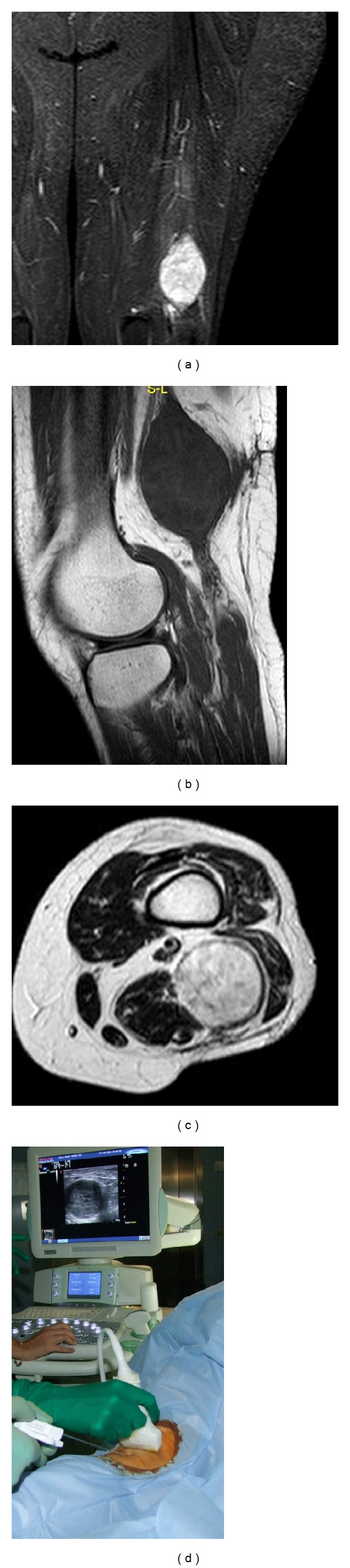
Coronal MRI STIR (a), sagittal MRI T2 (b), and axial MRI T1 (c) scans of the left thigh show homogeneous intratumoral hypointensive signal on T1 and hyperintensive signal on T2. A 14-gauge ultrasound-guided soft tissue percutaneous biopsy (d) of the left tight. Diagnosis: schwannoma.

However, ultrasound-guided soft tissue percutaneous biopsies (Figure 3) have been reported to have a high rate of accuracy [4]. In fact, the real time multiplanar visualization of the needle provides not only a safe procedure, visualizing vital structures, but also selective sampling of areas within the tumor, avoiding cystic or necrotic areas [4]. The biopsy needle is inserted along the same longitudinal plane as the ultrasound guidance to aid visualization of the needle [4].

In summary, open biopsy seems to be the most accurate modality, but there is not strong enough evidence to suggest one biopsy technique over another [19]. This lack of evidence should encourage investigators to analyze the diagnostic accuracy of these different biopsy techniques. 

## 3. Conclusion

The goal of biopsy is to obtain diagnostic tissue while minimizing morbidity, limiting potential tumor spread, and avoiding interference with future treatments [1]. The current literature does not allow to clarify what is the optimal biopsy technique for the diagnosis of bone and soft tissue tumors. Because of the low risk of contamination and the low cost, a percutaneous core-needle biopsy is usually preferred to an open surgical biopsy. In addition, the use of imaging-guided musculoskeletal biopsy increases diagnostic accuracy and reduces risk of complications. However, if a percutaneous biopsy is nondiagnostic, a small incisional biopsy should be performed. Therefore, successful of musculoskeletal biopsy depends by a close collaboration between the orthopaedic oncologist and the pathologist who can determine when a secondary biopsy is necessary.
